# Noradrenaline effects on social behaviour, intergroup relations, and moral decisions

**DOI:** 10.1016/j.neubiorev.2016.03.031

**Published:** 2016-07

**Authors:** S. Terbeck, J. Savulescu, L.P. Chesterman, P.J. Cowen

**Affiliations:** a Department of Psychology, University of Plymouth, Drake Circus, Plymouth PL48AA, United Kingdom; b Oxford Centre for Neuroethics, University of Oxford, Littlegate House, St Ebbes St, Oxford OX1 1PT, United Kingdom; c Department of Psychiatry, University of Oxford, Warneford Hospital, Oxford OX3 7JX, United Kingdom; d Cygnet Hospital Stevenage, Graveley Road, Stevenage SG1 4YS, United Kingdom

## Abstract

Recent research has begun to elucidate the neural basis of higher order social concepts, such as the mechanisms involved in intergroup relations, and moral judgments. Most theories have concentrated on higher order emotions, such as guilt, shame, or empathy, as core mechanisms. Accordingly, psychopharmacological and neurobiological studies have investigated the effects of manipulating serotonin or oxytocin activity on moral and social decisions and attitudes. However, recently it has been determined that changes in more basic emotions, such as fear and anger, might also have a significant role in social and moral cognition. This article summarizes psychopharmacological and fMRI research on the role of noradrenaline in higher order social cognition suggesting that indeed noradrenergic mediated affective changes might play key – and probably causal – role in certain social attitudes and moral judgments. Social judgments may also be directly influenced by numerous neurotransmitter manipulations but these effects could be mediated by modulation of basic emotions which appear to play an essential role in the formation of social concepts and moral behaviour.

## Highlights

• The involvement of noradrenaline in moral and social judgments is reviewed.
• Noradrenergic transmission is causally involved in implicit racial biases, racial face perception, as well as increasing social harm aversion.
• Fear and anger – mediated by limbic circuit brain activation – might mediate moral and social decisions and acts.

## 1 Introduction

*“The lower levels in the neural edifice of reason are the same ones that regulate the processing of emotions and feelings, along with the body functions necessary for an organism's survival. In turn, these lower levels maintain direct and mutual relationships with virtually every bodily organ, thus placing the body directly within the chain of operations that generate the highest reaches of reasoning, decision making, and, by extension, social behaviour and creativity. Emotion, feeling and biological regulation all play a role in human reason.”*(Damasio, 1994; p. Xvii).

There has been a long history of research, demonstrating that basic emotions, such as anger, fear, and happiness, can have an effect on reasoning (Pham, 2007), including working memory capacity (i.e., Darke, 1988), categorisation of stimuli (Ibsen et al., 1992), problem-solving (Isen et al., 1987), self-control (Fry, 1975) and risk taking behaviour (Johnson and Tversky, 1983). But basic emotions may also influence attitudes towards other races and decisions about what is morally right and wrong. Indeed, Noradrenaline (NA) based automatic emotional arousal may play a key role in the neurobiology of higher order psychological processes. Findings following this line of research might be understood within the framework of the somatic marker hypothesis (Levy et al., 2014), according to which somatic states influence – and often positively aid – explicit responses, and decisions (Damasio, 1994). For example noradrenergic beta receptors in the limbic brain are highly associated with emotional learning and emotion processing (Pitman and Delahanty, 2005), and key brain limbic regions proposed to mediate somatic markers (Bechara et al., 2005) might contribute to moral and social judgments. However, recent research in moral and social psychology has mostly focused on secondary emotions – social emotions – such as guilt and empathy. It has been stated that secondary emotions, particularly moral emotions differ from primary emotions in that they are often linked to the welfare of society or other individuals (Haidt, 2003). Moll (2008) suggested that moral emotions typically include guilt, pity, embarrassment, shame, pride, awe, contempt, indignation, and gratitude. It has been argued that moral emotions might be elicited in response to violations of social norms and stereotypes that code for individual attitudes and beliefs (Nichols, 2003). In his review, Pham (2007) concluded that secondary emotions (such as guilt, shame, gratitude) have a function in promoting socially and morally desirable behaviour. Further, Eisenberg (2000) suggested that moral emotions play a crucial role in helping people to evaluate moral features, motivate moral behaviour, and suppress immoral acts. However, primary emotions, and specifically noradrenergic mediated automatic emotional arousal, linked to fear and anger might have an equal contribution, and be a core factor, for social and moral decisions. This article will review recent research on the effect of noradrenaline on moral and social decisions. We will describe the role of noradrenaline in higher order social processes, and discuss how such findings could contribute to a more complete neural model of moral and social judgments.

## 2 Biochemistry of noradrenaline

Early research demonstrated that fearful stimuli elicit the “flight or fight” response in animals and humans; a response which is caused by sympathetic nervous system activation (Weiss, 1971), and which is mediated by adrenaline and noradrenaline (NA) release (Ax, 1953). NA belongs to the chemical class of catecholamines and is synthesised from the amino acid precursor phenylalanine and tyrosine. In the brainstem noradrenergic neurons, which mainly function to control blood pressure and flexor muscles, populate the medulla oblongata and the dorsal vagal nucleus with projections to the spinal cord (Leonard, 2003). A high density of noradrenergic cells bodies can also be found in the locus coeruleus which innervates the thalamus, dorsal hypothalamus, hippocampus and cortex. The ventral noradrenergic bundle, caudal to the locus coeruleus, is connected to subcortical limbic regions. Peripherally, noradrenaline is part of the sympathetic nervous system, mediating physiological responses of stress and acute anxiety, such as dilatation of pupils and bronchioles, increased heart rate, constricted blood vessels, increased kidney renin secretion, and inhibited peristalsis.

In the face of a threat (either real or perceived) behavioural adaptations to maintain homeostasis are activated with the NA system being the most prominent modulator (Morilak et al., 2005). Numerous studies in animals, but also in humans have shown activation of NA in response to stressful stimuli such as immobilisation, loud noise, immune challenge, electric shock, hypotension, or cold exposure (see Morilak et al., 2005 for a review). For example, early studies on experimental animals have found that various stressors (e.g., extreme temperatures, foot shock) significantly and selectively reduced brain NA concentration, whilst dopamine and serotonin levels seemed to be unaffected (e.g. Bliss et al., 1968, Corrodi et al., 1968). For example, exposure to inescapable shock/stress led to reduced central noradrenaline (Weiss, 1971). Additionally, Galvin (1985) found that acute stressors elevated MHPG-SO4 (a metabolite of NA) levels in limbic regions. This net increase was also correlated with plasma cortisol elevation. Thus, in his early review, Galvin (1985) concluded that the evidence supported the notion that central noradrenaline is involved in stress responses, stress pathology, and consequences of stress exposure. More recently, Morilak et al. (2005) reviewed their studies on animal responses to acute stress. They found that stress activated NA release in a number of brain regions including the central and medial amygdala, lateral bed nucleus of the stria terminalis, medial prefrontal cortex, and lateral septum. Using microinjections in those regions to administer NA antagonistic drugs they determined NA-mediation of a number of stress related behaviours in rats, such as activation of defensive burying and reduction of social interaction. The authors argue that neurobiological changes in those regions might contribute to psychiatric disorders such as anxiety, post-traumatic stress disorder (PTSD), or depression.

Levels of NA activity can be manipulated (i.e., reduced or enhanced) with various psychotropic drugs acting on adrenoreceptors. Adrenoreceptors (in α- and β form) are metabotropic G-protein-coupled receptors. Within the beta receptors β1, β2, and β3, subtypes can be determined. For example, propranolol, a centrally and peripherally acting beta receptor antagonist of both β1 and β2 receptors was originally developed for hypertension, but due to its inhibitory effect on emotional arousal also is a widely used treatment for stress, acute anxiety, and performance anxiety (e.g., Tyrer and Lader, 1974). Additionally, recent research has suggested that, by reducing the consolidation of emotional memory, propranolol might also be effective in preventing the development of PTSD, whether by being administered before exposure to a potentially traumatic situation or immediately afterwards (Mills and Dimsdale, 1991, Pitman and Delahanty, 2005, Stein et al., 2007).

Buffalari and Grace (2007) researched the effect of propranolol on the basolateral amygdala using single unit neuronal recordings in rats. The authors found that NA enhances tonic excitatory effects via beta receptors in the basolateral amygdala, whilst beta adrenergic blockade lead to a decrease in spontaneous firing rate. Several studies have shown that single doses of propranolol also influence emotional processing in humans (Hurlemann et al., 2010, Van Stegeren et al., 2005) and that propranolol reduces physiological markers of high arousal (heart rate, potentiated startle response) following emotion-evoking stimuli (Mills and Dimsdale, 1991, Davis et al., 1993). In addition, functional neuroimaging studies have shown that propranolol leads to a reduction in amygdala responses to emotional pictures or emotional facial expressions (Hurlemann et al., 2010, Van Stegeren et al., 2005).

In contrast, reboxetine is an example of a selective noradrenaline re-uptake blocker. This drug is used as antidepressant medication and increases synaptic levels of NA by blocking the re-uptake of NA back into the presynaptic terminal, thereby indirectly activating both postsynaptic alpha and beta receptors. Therefore, in terms of the receptors whose actions are influenced, reboxetine will have a broader spectrum of action than propranolol (Leonard, 2003). Single doses of reboxetine in healthy volunteers have been shown to produce a positive bias in implicit emotional processing in terms of facial expression recognition and emotional memory (e.g. Harmer et al., 2004, Norbury et al., 2008). Acute reboxetine administration increases heart rate, presumably through stimulation of cardiac β1 receptors (Leonard, 2003). It is therefore likely that reboxetine will increase basic emotional arousal.

Previous research has also given evidence for NA being involved in social behaviour, Vanderschuren et al. (1997) reviewed the neurobiology of social play in rats. The authors discussed that whilst dopamine and opioids were involved in the reward aspect of social play, noradrenergic systems were also involved in the attentional processes relevant to social play behaviour. Vanderschuren et al. (1997) argue that social play in animals might have the function of establishing social organisation within groups and to inhibit aggression and increase group stability. Previous studies have found that amphetamine, which stimulates catecholamine release, depressed social play (e.g., Beatty et al., 1984), but increased social investigation (Beatty et al., 1982). Propranolol administration also decreased social play (Beatty et al., 1984), although Vanderschuren et al. (1997) argued that the role of noradrenaline in social play was still not understood and that the effects seen might depend on the particular NA receptors (i.e., alpha 1 versus alpha 2 versus beta receptors) targeted. More recently, Achterberg et al. (2016) investigated the differential effect of dopamine and NA in social play in rats, determining that dopamine was more strongly associated with motivation for play whilst NA enhancement negatively modulated both motivation as well as expression of social play. Furthermore, Achterberg et al. (2015) determined that infusion of methylphenidate and atomoxetine into prefrontal and limbic brain areas inhibited social play (via increased NA activity), but not social exploratory behaviour, further supporting the modulatory role of NA in the development of social play behaviour. Vanderschuren et al. (2008) also found that methylphenidate abolished social play in rats. As methylphenidate and atomoxetine are first-choice treatment for attention-deficit/hyperactivity disorder (ADHD) in children, such effects are potentially important in social development and are worthy of careful assessment in clinical studies.

## 3 Noradrenaline and intergroup attitudes

Research on prejudice and stereotypes started in the 1920s and 1930s, a time when there was overt racial conflict and legally sanctioned racial discrimination in the United States. However, biases continue to operate in contemporary society, especially on measures of implicit attitude (i.e., Greenwald et al., 1998). Compared to explicit prejudice and stereotypes about out-group members, individuals also display implicit associations towards out-group members that might be automatic and occur even with a sincere explicit belief in equality (Nosek et al., 2007, Terbeck et al., 2012a). Indeed correlations between explicit and implicit out-group evaluations are found to be usually weak (∼r < 0.15) (Nosek et al., 2007), which suggest that they may have distinct underlying neurobiological mechanisms (De Houwer et al., 2009).

The implicit association test (IAT) (Greenwald et al., 1998) is a widely used method to assess implicit attitudes (Nosek et al., 2007). In this item-sorting, response time based task, participants perform a categorisation task by sorting positive/negative words and pictures of faces from black/white individuals. The IAT effect is determined by latency differences in response times to bias congruent (good white/bad black) versus incongruent (good black/bad white) trials. Extensive evidence shows that the IAT can be regarded as a reliable measure of implicit attitudes towards social out-groups, whether based on race, sexual orientation, gender, or political preference (Nosek et al., 2007). A meta–analysis further suggested that the IAT is a better predictor of some forms of discrimination against out-group members than explicit measures (Poehlman et al., 2004). Several studies, specifically fMRI studies have suggested that, implicit “prejudice” involves a stronger emotional component than explicit prejudice (Lieberman, 2005, Wheeler and Fiske, 2005).

One of the earliest neuroscientific investigations of racial attitudes was conducted by Phelps et al. (2000). These researchers used fMRI to assess the blood-oxygen-level-dependent (BOLD) responses in association with white, compared to black, unfamiliar male faces (participants were white Americans). Outside the scanner, they also assessed racial attitudes of the participants using implicit and explicit measures. Importantly, the authors found that the magnitude of the BOLD response to black versus white faces in the amygdala was significantly correlated with the implicit, but not the explicit, measure of racial bias. Subsequent research has demonstrated that other brain areas, including the dorsolateral prefrontal cortex (dlPFC) and the anterior cingulate cortex (ACC) might contribute to mechanisms reviewing the initial evaluation (Cunningham et al., 2004, Knutson et al., 2007, Richeson et al., 2003, Stanley et al., 2008).

**Fig. 1:**
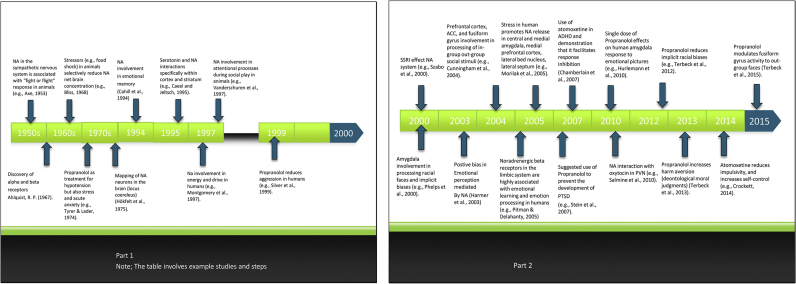
Chronological representation of the evolution of the knowledge of Noradrenaline effects.

Recently, our own research confirmed previous theorizing on the role of primary emotions, in implicit racial attitudes (Terbeck et al., 2012a, Terbeck et al., 2012b). In this psychopharmacological study, healthy volunteers of white ethnic origin, received a single oral dose of the propranolol (40 mg). Participants completed an explicit measure of prejudice and the racial IAT 1–2 h after propranolol administration. Importantly, relative to placebo, propranolol reduced the implicit racial bias, without affecting the explicit racial prejudice measure. These results indicate that β‐adrenoceptors play a role in the expression of implicit racial attitudes suggesting that noradrenaline related emotional mechanisms may mediate racial biases. Furthermore, using fMRI following the same pharmacological manipulation, we found support for the suggestion that central automatic emotional arousal might be causally involved in implicit racial biases (Terbeck et al., 2015). In this study 40 participants also received propranolol or placebo in a double blind manner. Participants then viewed unfamiliar black or white faces during the fMRI scan and subsequently completed the racial implicit association test. Propranolol reduced the IAT score and led to reduced activity for black compared to white faces in fusiform gyrus (See Fig. 1). Furthermore, time course analysis of the fusiform gyrus activity demonstrated that sensitization to the viewing of black faces, which was observed with placebo, was diminished with propranolol. We suggested that the racial face perception at early stages was thus modulated by basic noradrenergic activity. Indeed, our research supports previous accounts suggesting that face processing areas (i.e., fusiform gyrus) might mediate the processing of ingroup versus outgroup faces (e.g., Golby et al., 2001).

In conclusion, most neuroscience studies, fMRI research (Cunningham et al., 2004, Knutson et al., 2007, Richeson et al., 2003, Terbeck et al., 2012b) and recent psychopharmacological studies (Terbeck et al., 2012b) have supported the theory that basic emotional arousal, such as fear or aggression, possibly mediated by noradrenergic activation, might be causally relevant for implicit social attitudes.

## 4 Noradrenaline and moral judgment

The earliest investigations of morality can be found in Plato’s approaches over 2000 years ago (Casebeer and Churchland, 2003). Casebeer and Churchland, (2003) defined moral reasoning as “… a series of acts that result in a conclusion about what one ought to do or think”. To research human moral decision-making, most researchers use theoretical moral judgments, such as the trolley dilemma: *You are at the wheel of a runaway trolley quickly approaching a fork in the tracks. On the tracks extending to the left is a group of five railway workmen. On the tracks extending to the right is a single railway workman. If you do nothing, the trolley will proceed to the left, causing the deaths of the five workmen. The only way to avoid the deaths of these workmen is to hit a switch on your dashboard that will cause the trolley to proceed to the right, causing the death of the single workman. How morally acceptable is it to hit the switch in order to avoid the deaths of the five workmen?*

This dilemma involves harming one as a side-effect of saving five. In contrast, the second “up close and personal” version involves pushing a person in front of the trolley as a means to stopping it killing 5 people further down the track. This harm is sacrificial and involves using the person killed as a means to saving the five. Typically people switch in the first “impersonal” version to save the five, but refuse to kill the one in the second to save the five. According to emotivists, moral decisions are expressions of pro or con attitudes. Also sentimentalism supports the idea that emotion plays a central, causal role in moral judgments (Nichols and Mallon, 2005). For example, Gibbard, (1990) argued that feeling guilty about an action is the central emotional response in guiding moral choice.

Previous research has already suggested that there might be associations between primary emotional arousal and moral judgments. For example, Carlson and Miller (1987) found that positive mood enhanced pro-social behaviour. And Scher (1997) demonstrated that anger and sadness were linked to perceptions of injustice and immorality. In addition Wheatly and Haidt (2005) found that subjects, who were hypnotized to feel disgust, made more severe moral judgments. Haidt and Bjorklund (2008) suggested that moral decisions are the product of quick and automatic intuitions, whilst conscious reasoning on the other hand occurred as a post-hoc justification of already preferred options chosen by intuition. Furthermore, Moretto et al. (2010) found that healthy individuals, but not patients with ventromedial prefrontal cortex (vmPFC) damage, generated skin conductance responses (a somatic marker of affective arousal), before making judgments in personal dilemmas. This anticipatory skin conductance response was negatively correlated with the frequency of utilitarian judgments in healthy individuals, suggesting involvement of somatic emotional arousal in moral decision-making, that is the greater the affective response the less the chance of making a utilitarian judgment.

We recently investigated the role of noradrenergic transmission in moral decision-making (Terbeck et al., 2013). In a double-blind study 40 participants received propranolol or placebo before judging moral acceptability in theoretical moral dilemmas. We found that propranolol made participants more likely to judge sacrificial actions as morally unacceptable. Additionally, we only found this effect in dilemmas where harms were ‘up close and personal’ (i.e., those involving more active and direct harmful action). Previously, Greene et al. (2001) suggested that deontological moral judgments (e.g., judgments that determine the moral worth of action or rules, regardless of the consequences) are driven by emotion, whilst utilitarian judgments (i.e., judgments of morality that are determined by the resulting outcome) are caused by cognitive processes overriding initial emotional judgments. Additionally, Greene (2008) suggested that emotional and cognitive processes compete, leading to conflict (as reflected by for example activation of the anterior cingulate cortex).

Our results with propranolol, however, (Terbeck et al., 2013) are contrary to this theory, suggesting that general physiological arousal (at least that associated with NA activation) is not likely to play a causal role in deontological judgments. We suggested however that propranolol administration might have led to an increase in aversion to harming others directly (that is, in sacrificial moral dilemmas). Indeed, this is in line with extensive evidence that propranolol reduces aggression (Greendyke et al., 1986, Silver et al., 1999), supporting the idea that basic emotions − such as harm aversion and aggression − might contribute to such moral choices. Furthermore, other pharmaceuticals, such as atomoxetine (general cognitive enhancers), commonly used to treat attention deficit hyperactivity disorder, might have stronger effect on aggression and harmful social behaviour by reducing impulsivity, and thus increasing self-control of anger and fear responses (Crockett, 2014).

## 5 Noradrenaline in higher order social concepts: comparing the effect

**Fig. 2:**
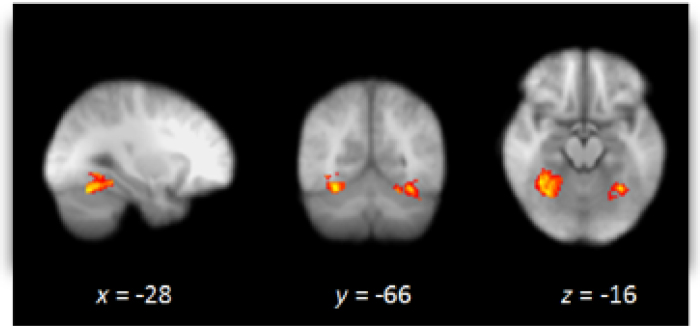
Whole Brain image under placebo versus propranolol to black versus white faces. Whole brain image depicting greater activation under placebo versus propranolol to black versus white faces. Image thresholded at *Z *= 2.3, *p* > 0.05, corrected. Images are in radiological format (right brain on *left*).

Source: from Terbeck et al. (2015).

In the previous sections we have discussed research on the involvement of NA in higher order social and moral judgments. Indeed the knowledge of the effect of NA has greatly advanced over the last decade. Fig. 2 gives an overview of the evolution of knowledge of NA and its effects on human behaviour.

However the action of NA cannot be viewed in isolation; instead its interactions with other neurotransmitters need to be considered. In the following, we will describe the activity of two other key neurotransmitters − namely serotonin and oxytocin − which have also been shown to influence social behaviour in humans. We will then describe possible interaction mechanisms of these different neurotransmitter systems. Serotonin levels are increased with selective serotonin reuptake inhibitors (SSRIs), which are commonly prescribed for the treatment of depression and a wide range of anxiety disorders. SSRIs block the re-uptake of serotonin in the presynaptic nerve terminal thereby increasing its activity at the synapse and indirectly stimulating all post-synaptic serotonin receptor subtypes. In some patients, SSRIs alleviate depressed mood and reduce excessive anxiety, which has been suggested to be the result of increasing cognitive control over basic limbic processes (Levy et al., 2014). Similar to effects observed with propranolol, SSRIs have also been shown to affect moral behaviour. For example, participants taking SSRIs were more cooperative and less critical (Knutson et al., 2007) and more socially affiliative towards others (Tse and Bond, 2002). Furthermore, Crockett et al. (2010) found that tryptophan depletion (which reduces net brain serotonin concentration) led to increased rates of rejection of unfair offers. More recent work has suggested that potentiating serotonin increased deontological moral judgment, which the authors explained as an effect of increased aversion to causing direct harm to others (Crockett et al., 2010). Interestingly, further analysis revealed that this effect was driven by an increase in harm aversion only in subjects who were already highly empathetic prior to administration of the drug (Levy et al., 2014).

Another neurotransmitter that has been suggested to have effect on levels of empathy, bonding, and love is the hormone and neurotransmitter, oxytocin. Oxytocin facilitates birth and breastfeeding in humans, but also moral behaviour and intergroup relations. Numerous research studies in humans and animals have suggested that oxytocin mediates pair bonding, maternal care, and general pro-social behaviour (e.g., Insel and Fernald, 2004). For instance Kosfeld et al. (2005) found that the administration of oxytocin led to more trusting behaviour in humans, that is, investigators entrusted the trustee with a significantly greater amount of money (Kosfeld et al., 2005). De Dreu et al. (2010) were the first to examine the effects of oxytocin on moral behaviour and intergroup relations. In a series of moral dilemmas, the authors found that with placebo, the race of a person who would be directly harmed to save many others did not have a significant effect on the moral decision. However, in the oxytocin group, race did play a role in the moral decisions, making subjects more averse to harming one individual to save the lives of others. Specifically, the authors found that participants administered oxytocin were significantly more likely to sacrifice a different-race individual in order to save a group of race-unspecified others than they were to sacrifice a same-race individual in the same circumstances. Further research by De Dreu et al. (2010) also suggested that oxytocin might promote empathy in pro-social behaviour but maybe only for in-group members, reducing pro-social behaviour towards the out-group.

The above demonstrates that in addition to noradrenaline, other neurotransmitters can also have an effect on moral decisions and intergroup relations. As demonstrated above, different pharmaceutical interventions, with different effects on experience, can produce seemingly similar changes to moral behaviour. For instance, while Crockett et al. (2010) found that reduction of serotonin activity increases in deontological moral decisions (unwillingness to sacrifice one to save five), we found that reduction in noradrenaline transmission (Terbeck et al., 2013) produced the same effect. It is indeed difficult to determine if both effects are mediated by similar neural mechanisms, but recent pharmacological fMRI supports the view that different neural circuitry may be involved (Terbeck et al., 2015). For example, in our fMRI study of other race faces we found that noradrenaline particularly affected neural activity in the thalamus and fusiform gyrus, suggesting that noradrenaline might be involved in processes mediating the initial perception and evaluation of emotional stimuli. Furthermore, NA is suggested to affect basic primary emotional arousal such as fear and aggression within the limbic system (Leonard, 2003). On the other hand, the effect of oxytocin on social/affiliative responses has been suggested to involve systems in the ACC, insula, and amydgala (e.g., De Dreu et al., 2010, Insel and Fernald, 2004, Kosfeld et al., 2005). Serotonin effects might be mostly involed in cognitive control over limbic processes, suggested to play a key role in controlling mechanisms of basic emotional arousal giving rise to secondary emotions, such as guilt and shame (for a review see for example Adolphs, 2009).

However, NA pathways are widely distributed and the effect of NA manipulation onmoral and social decision making should also be viewed in the context of interactions with serotonin and oxytocin neurons. Indeed interactions between NA and serotonin have long thought to be important in the pathophysiology and treatment of mood disorders (Brunello et al., 2003) For example, chronic administration of SSRIs increases the density of NA beta1 receptors in rat brain (Pälvimäki et al., 1994). Also, SSRIs have been shown to lead to long term adaptive changes in the NA system in humans (e.g., Szabo et al., 2000).

In their review, Cassel and Jeltsch (1995) argued that several brain regions including the hippocampus, cortex and striatum might be critical for NA and serotonin interactions. It is well established that NA and serotonin can modulate numerous cognitive processes including learning, memory, sleep, arousal, and anxiety (Montgomery, 1997, Brunello et al., 2003) It might thus also be suggested that NA affects social behaviour by mediating attentional processes directly but also by influencing serotonin pathways involved in modifying social judgements in cortical regions. NA might also shape social affiliation and intergroup relations via interactions with oxytocin. As stated above, oxytocin is involved in the regulation of reproductive function, social and sexual behaviours but also stress responses (Salmina et al., 2010). There is much evidence suggesting an interaction between NA and oxytocin, also with regards to regulating social behaviour. For example, Salmina et al. (2010) described evidence suggesting that oxytocin release was regulated by NA in the hypothalamic-neurophysical system, specifically including the supraoptic nucleus and the paraventricular nucleus (PVN). The authors argued that NA activates presynaptic glutamate interneurons closely located around PVN and therewith indirectly causes oxytocin release. Furthermore, Onaka et al. (2012) also reviewed evidence that oxytocin release was regulated in part by NA activity, and thereby both neurotransmitters modulated functions such as anxiety, energy metabolism and attachment. Stress − such as that occasioned by public speaking − leads to oxytocin release, indicating that oxytocin might have an anxiolytic effect (Onaka et al., 2012). Interactions of oxytocin with NA have also been located in projections in the medulla oblugata, whilst similar interactions in the amydala might be important in the regulation of social behaviour (Onaka et al., 2012). It could thus be argued that NA mediated emotional arousal is involved in implicit biases though interaction with oxytocin, which promotes in-group favoritism. Specifically, basic emotion processing (via NA) might regulate complementary mechanisms (via oxytocin) that produce ingroup favoritism and social bonding within one's own social group.

In conclusion, we have reviewed the influence of noradrenaline on higher order psychological processes, suggesting how different mechanisms might mediate the effects. We suggest that fMRI studies involving pharmacological manipulation may elucidate the role of different brain regions and neurotranmitter interactions involved in social and moral decisions. Such a combined approach is a potentially fruitful line of enquiry.
